# Editorial: Molecular Mechanisms in Pulmonary Hypertension and Right Ventricle Dysfunction

**DOI:** 10.3389/fphys.2018.01777

**Published:** 2018-12-10

**Authors:** Harry Karmouty-Quintana, Christophe Guignabert, Grazyna Kwapiszewska, Mark L. Ormiston

**Affiliations:** ^1^Department of Biochemistry and Molecular Biology, University of Texas Health Science Center at Houston, Houston, TX, United States; ^2^Institut National de la Santé et de la Recherche Médicale UMR_S 999, Le Plessis-Robinson, France; ^3^Université Paris-Sud and Université Paris-Saclay, Le Kremlin-Bicêtre, France; ^4^Ludwig Boltzmann Institute for Lung Vascular Research, Graz, Austria; ^5^Otto Loewi Research Center, Division of Physiology, Medical University of Graz, Graz, Austria; ^6^Department of Biomedical and Molecular Sciences, Queen's University, Kingston, ON, Canada; ^7^Department of Medicine, Queen's University, Kingston, ON, Canada; ^8^Department of Surgery, Queen's University, Kingston, ON, Canada

**Keywords:** pulmonary hypertension (PH), pulmonary hypertension (PH) due to lung diseases and/or hypoxemia, endothelial cell (EC), right ventricle (RV) function, vascular smooth muscle

Pulmonary hypertension (PH) is a hemodynamic condition with multiple etiologies that is defined as a mean pulmonary arterial pressure (mPAP) of at least 25 mmHg at rest measured during right heart catheterization, leading to right heart failure and death (Simonneau et al., 2013; Galiè et al., 2015). PH can result from pre-capillary (arterial) or post-capillary (venous) pathomechanisms. Group 1 PH corresponds to pulmonary arterial hypertension (PAH), which is characterized by pre-capillary PH (mPAP greater or equal to 25 mmHg with a normal pulmonary capillary wedge pressure ≤ 15 mmHg) due to major pulmonary arterial remodeling. PH associated with chronic lung diseases such as idiopathic pulmonary fibrosis (IPF) is classified as Group 3 PH. In all of these forms, the diagnosis of PH is strongly associated with increased morbidity and mortality and in the vast majority of cases, PH remains a progressive, incurable disorder.

This research topic aims at enhancing our understanding of the mechanisms that contribute to the pathophysiology of Group 1 or Group 3 PH and right ventricle hypertrophy, as well as the development of novel therapeutics for PH. In this research topic, Segura-Ibarra et al. discussed innovative nano-therapeutics as potential therapies to treat Group 1 and Group 3 PH. Also, restoration of iron homeostatic balance could have the potential for therapeutic options in PAH are proposed by Ramakrishnan et al. in their review. The authors described that defects in iron homeostasis are associated with vascular remodeling and PAH.

A key cell in the pathogenesis of PH is the pulmonary endothelial cell (EC) (Guignabert et al., 2015; Huertas et al., 2018; Thenappan et al., 2018). At the interface between the bloodstream and the vessel wall, the pulmonary endothelium has a very significant role in controlling barrier integrity and function (Huertas et al., 2018). Injury or insult to ECs is a known mechanism that can lead to the development of PH (Guignabert et al., 2009, 2016; Thenappan et al., 2018). In this research topic, Fazakas et al. demonstrate that the oral multi-target tyrosine kinase inhibitor, dasatinib at high doses alters EC integrity causing changes in cell morphology and reorganization of the actin cytoskeleton consistent with increased pulmonary pressure in isolated perfused and ventilated rat lungs. This study also demonstrates that some of the effects of dasatinib are modulated by Rho-kinase activation, a signaling that is abnormally activated in both Group 1 and Group 3 settings (Guilluy et al., 2009; Collum et al., 2017b). Although parts of these findings are in line with previous studies demonstrating that dasatinib-induced endothelial cell dysfunction is reversible following withdrawal (Phan et al., 2018), it appears clear that this phenomenon cannot be sufficiently explained by ROCK activation or Lyn inhibition alone (Phan et al., 2018). Further work is therefore needed to identify the mechanisms underlying dasatinib induced lung vascular toxicity and PH predisposition (Guignabert et al., 2016). Hypoxia-induced EC injury is another key event that is associated with vascular remodeling in PH (Xu and Erzurum, 2011). In this research topic Pi et al. demonstrate that attenuation of the endothelial production of connective tissue growth factor (CTGF) decreased cdc42 activity and protected against hypoxia induced PH and bleomycin (BLM)-induced lung fibrosis and PH.

Enhanced hypoxic-adenosinergic axis is an important feature of Group 3 PH (Garcia-Morales et al., 2016) that is mediated through activation of the adenosine A2B receptor [ADORA2B (Karmouty-Quintana et al.)]. In these studies, genetic deletion of ADORA2B in mice or treatment with an ADORA2B antagonist was able to inhibit BLM-induced fibrosis and PH. Herein Mertens et al. uncouple the role of vascular smooth muscle cell in vascular remodeling, demonstrating that conditional deletion of ADORA2B from smooth muscle cells protects mice from the development of BLM-induced PH without altering fibrosis. These studies demonstrate that therapies aimed at targeting ADORA2B may be effective at treating Group 3 PH where there is an urgent need for new treatments (Hoffmann et al., 2014; Collum et al., 2017a). Rathinasabapathy et al. evaluated the role of the renin-angiotensin system in the development of PH associated with lung fibrosis. In these studies, treatment with either recombinant human Angiotensin Converting Enzyme 2 (rhACE2) (Rathinasabapathy et al.) or a selective Angiotensin II Type 2 (AT2) receptor agonist (Rathinasabapathy et al.) was able to attenuate both PH and fibrotic deposition induced by BLM. These effects were associated with a depletion of inflammatory cells, particularly macrophages, and were accompanied by improved right ventricle (RV) function.

The role of inflammation and fibrosis modulating right ventricular (RV) remodeling and dysfunction is explicitly addressed in this topic by three articles. In the first study, Tian et al. examined the impact of altered mitochondrial dynamics in RV fibroblasts on RV dysfunction and collagen deposition in monocrotaline challenged rats. The authors demonstrate that dynamin related protein-1 (Drp-1) inhibitors, including Mdivi-1 and P110, reverse mitochondrial network fragmentation, cellular hyperproliferation and elevated collagen production in RV fibroblasts from monocrotaline challenged rats *in vitro*. However, the *in vivo* administration of P110 was not sufficient to prevent PH and pathological RV remodeling in response to monocrotaline challenge. To accompany this study, a pair of reviews by Sydykov et al. and Dewachter and Dewachter discuss the role of inflammatory mediators in maladaptive RV remodeling and dysfunction. These reviews identify the major cytokines, chemokines, and immune cell subsets that have been linked to RV dysfunction and failure in both humans and animal models of RV overload. Interestingly, both studies highlight previous reports demonstrating more severe RV impairment in patients with PAH secondary to systemic sclerosis (SSc) when compared to patients with idiopathic disease and comparable afterload. These works also examine the prospect of therapeutic strategies that aim to improve RV function through the targeting of inflammatory processes, as well as the potential use of inflammatory factors as biomarkers of RV dysfunction.

Currently the golden standard for diagnosis of PH patients is right heart catheterization. However, for monitoring, follow-up or sub-classification (endotyping) of the patients, application of biomarkers could be extremely useful. Identification of valid biomarkers are a crucial step toward precision medicine. In the current issue, three articles have focused on the importance of the biomarkers in PH. Grünig et al. tested the idea that circulating miRNA are associated with PH and that their levels depend on exercise and oxygen-therapy. Circulating miRNAs that control muscle and erythrocyte function (miR-22-3p, miR-21-5p, miR-451a) were decreased upon supervised exercise training or nightly oxygen intervention, pointing to a role as biomarkers of PH progression that are responsive to intervention. Indeed, miRNAs could be very promising biomarkers as they are very stable in bodily fluids such as blood (plasma and serum). In their review, Odler et al. described several biomarkers that have been associated with SSc-PAH. These biomarkers reflected endothelial physiology (e.g., vWF, endostatin), immune activation (e.g., CXCL4), extracellular matrix (e.g., osteopontin), metabolic changes (e.g., adipocytokines), or cardiac involvement (e.g., troponin T). They can range from peptides, cytokines, auto-antibodies up to miRNAs. Although most biomarkers were associated with diagnosis, disease severity, or progression, the authors point out that they rarely have been tested in a prospective studies using well-defined patient cohorts. Sydykov et al. in their review highlighted how inflammatory cells and their mediators can serve as biomarkers of RV remodeling and dysfunction. Higher numbers of macrophages, mast cells and leukocytes as well as cytokines/chemokines such as Il-6, TNF-α, CXCL10, CXCL12 correlated with worsened RV functions such as RV end-diastolic diameter, mean right atrial pressure, and cardiac index. However, performance of these biomarkers in clinical applications requires further validation. In conclusion, this special issue identified and discussed several important target molecules, which could help in the development of new potential diagnostic and therapeutic options. However, further research is necessary to pursue innovative biomarkers and subsequent translational studies are needed to attenuate the high morbidity and mortality associated with all forms of PH.

## Author Contributions

All authors listed have made equal substantial, direct, and intellectual contribution to the work, and approved it for publication.

### Conflict of Interest Statement

The authors declare that the research was conducted in the absence of any commercial or financial relationships that could be construed as a potential conflict of interest. The handling editor declared a shared affiliation, though no other collaboration, with one of the authors MO at time of review.
